# Unremitting pro‐inflammatory T‐cell phenotypes, and macrophage activity, following paediatric burn injury

**DOI:** 10.1002/cti2.1496

**Published:** 2024-03-08

**Authors:** Donna Langley, Kate Zimmermann, Emma Krenske, Giorgio Stefanutti, Roy M Kimble, Andrew JA Holland, Mark W Fear, Fiona M Wood, Tony Kenna, Leila Cuttle

**Affiliations:** ^1^ School of Biomedical Sciences, Faculty of Health Queensland University of Technology (QUT) South Brisbane QLD Australia; ^2^ Centre for Immunology and Infection Control (CIIC) QIMR Berghofer Medical Research Institute, Queensland University of Technology (QUT) Brisbane QLD Australia; ^3^ Centre for Biomedical Technology (CBT) Queensland University of Technology (QUT) Kelvin Grove QLD Australia; ^4^ Department of Paediatric Surgery, Urology, Burns and Trauma Children's Health Queensland, Queensland Children's Hospital South Brisbane QLD Australia; ^5^ The Children's Hospital at Westmead Burns Unit, Department of Paediatrics and Child Health, Kids Research Institute Sydney Medical School, The University of Sydney Sydney NSW Australia; ^6^ Burn Injury Research Unit, School of Biomedical Sciences The University of Western Australia Perth WA Australia; ^7^ Burns Service of Western Australia Perth Children's Hospital and Fiona Stanley Hospital Perth WA Australia

**Keywords:** burns, cytokines, flow cytometry, inflammation, lymphocytes, paediatrics

## Abstract

**Objectives:**

The aim of this study was to characterise the dynamic immune profile of paediatric burn patients for up to 18 months post‐burn.

**Methods:**

Flow cytometry was used to measure 25 cell markers, chemokines and cytokines which reflected both pro‐inflammatory and anti‐inflammatory immune profiles. Peripheral blood mononuclear cells from 6 paediatric burn patients who had returned for repeated burn and scar treatments for > 4 timepoints within 12 months post‐burn were compared to four age‐matched healthy controls.

**Results:**

While overall proportions of T cells, NK cells and macrophages remained relatively constant, over time percentages of these immune cells differentiated into effector and proinflammatory cell phenotypes including Th17 and activated γδ T cells. Circulating proportions of γδ T cells increased their expression of pro‐inflammatory mediators throughout the burn recovery, with a 3–6 fold increase of IL‐17 at 1–3 weeks, and NFκβ 9–18 months post‐burn. T‐regulatory cell plasticity was also observed, and Treg phenotype proportions changed from systemically reduced skin‐homing T‐regs (CCR4^+^) and increased inflammatory (CCR6^+^) at 1‐month post‐burn, to double‐positive cell types (CCR4^+^CCR6^+^) elevated in circulation for 18 months post‐burn. Furthermore, Tregs were observed to proportionally express less IL‐10 but increased TNF‐α over 18 months.

**Conclusion:**

Overall, these results indicate the circulating percentages of immune cells do not increase or decrease over time post‐burn, instead they become highly specialised, inflammatory and skin‐homing. In this patient population, these changes persisted for at least 18 months post‐burn, this ‘immune distraction’ may limit the ability of immune cells to prioritise other threats post‐burn, such as respiratory infections.

## Graphical abstract

In this study, we found that circulating immune cells do not increase or decrease over time post‐burn, instead they become highly specialised, inflammatory and skin‐homing. Changes were observed in Th17 cells, γδ T cells, NKT‐like cells, and double‐positive CCR4^+^ CCR6^+^ Treg cells. In this paediatric patient population, these changes persisted for at least 18 months post‐burn, and this ‘immune distraction**’** may limit the ability of immune cells to prioritise other threats post‐burn, such as respiratory infections.

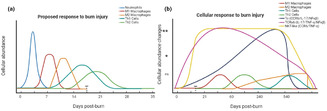

## Introduction

Burns injuries impact millions of people worldwide,^1^ at a mean total health care cost of US$88218 per patient.^2^ Burn injuries are particularly common in children, and children under the age of 5 years of age are especially vulnerable to burns. Children experience life‐long health impacts of burn injuries,^3^ and are at increased risk for chronic immune‐related conditions, including diabetes and respiratory infections.^4^ Other reported long‐term health deficits include persistent systemic inflammation, hypertrophic scarring, hypermetabolism, diminished vaccine and immune response and immune dysregulation.^5^, ^6^, ^7^, ^8^, ^9^ Infections, sepsis and multiple organ dysfunction syndrome^10^ are common for patients post‐burn.^11^, ^12^ Infection can delay wound healing,^13^, ^14^ contributing to scarring^15^ which impacts on a child's growth and development. The body's response to infection is a cellular response: anti‐microbials and antibodies are produced to mitigate the effect of the pathogens. There is no evidence of a known burn antigen or antibody, a single inflammatory marker or cell, to establish how the body deals with a burn injury. Historically, attempts have been made to identify the cause of immune dysregulation post‐burn injury as directed by a single burn toxin,^16^, ^17^ or by implicating immune suppression.^18^, ^19^, ^20^ More recent research is directed at understanding the systemic inflammatory immune response resulting from burn injury.^8^, ^21^, ^22^ However, the dynamics of immune cells and mediators within the first year of a burn remain largely unknown. The identification of immune and inflammatory mediators responsible for restoring homeostasis, or associated with excessive inflammation and remodelling post‐burn, would enable the identification of therapeutic targets and prognostic and diagnostic indicators of wound healing, which could be incorporated into management plans. The goal of this study was to characterise the cellular and cell‐mediated changes in the active phase of the burn event and observe their dynamic behaviours over 18 months, to develop an immune profile for paediatric burn patients.

## Results

### Patient details

Samples and patient data were collected from paediatric burns patients who presented to hospital with a burn or burn scar, between January 2020 and December 2021. This study consisted of six patients who had returned for repeated treatments and had peripheral blood mononuclear cells (PBMCs) collected over multiple timepoints (over 4+ timepoints) during the initial treatment of their burn. The patient samples were grouped into clinically relevant time points post‐burn, relating to early or late wound healing, scar formation and scar re‐modelling (1–3 weeks, 1–2 months, 3–8 months, 9–18 months), with a minimum of six samples per time point.

The age of the healthy controls was 3.5 ± 3 (mean ± SD) years of age, and the burn patients were 3 ± 2.5 years of age (Table 1). The burns ranged in size from 1.5 to 34% total body surface area. All burns were scald or contact, and deep to full thickness. The mean time to re‐epithelialisation was 58.5 ± 44.1 days post‐burn. Four of the six patients received antibiotics multiple times, except for patient 1 who received i.v. Cefazolin once on the day of the burn, but none afterwards. The patients with infections all tested positive to *Staphylococcus aureus*. Four of the six burn patients received skin grafts and five patients received microneedling or CO_2_ ablative laser therapy for their scars. In the clinical notes, the laser energy in mJ was often noted, and this was converted to relative scar thickness using the Lumenis laser ablation depth and energy conversion table.^23^ The Brisbane Burn Scar Impact Profile (BBSIP) is a patient or caregiver‐reported assessment of the quality of life for people with burn scars,^24^ and the worst Overall score is reported during the patient's treatment period. All patients had scarring which impacted on their daily life.

**Table 1:** Patient and burn injury details

	Controls				Burn patients					
Participant number	1	2	3	4	1	2	3	4	5	6
Age at 1st collection (years)	5	1	7	1	5	1	1	2	2	7
Gender	f	f	m	f	m	m	f	f	m	m
TBSA %					3	9	2	6	1.5	34
Mechanism of burn					Contact	Scald	Contact	Contact	Scald	Scald
Depth of burn injury					Deep	Deep	Full	Full	Full	Full
Re‐epithelialisation (DPB)					22	25	64	92	21	127
Grafted (DPB)					‐	21	‐	81	34	28
Infections (DPB)					‐	56	14	93	‐	302
Worst relative scar height (mJ or mm)					150 mJ/4 mm	120 mJ/3 mm	150 mJ/3 mm	nr	120 mJ/ 3 mm	150 mJ/ 4 mm
Worst BBSIP score					nr	1.62/5	1.25/5	2.38/5	1.6/5	1.6/5
Comments								HTS & contractures		

BBSIP, Brisbane Burn Scar Impact Profile; Deep, deep partial thickness burn; DPB, days post‐burn; f, female; Full, full thickness burn; HTS, hypertrophic scarring; m, male; mJ, milliJoules; mm, millimetres; nr, not recorded; TBSA, total body surface area.

### There were no significant shifts in the proportion of principal lymphocyte populations post‐burn

The proportions of principal lymphocyte populations were relatively unchanged over time post‐burn, and there were no large variations between control and burn, or burn time‐points (Supplementary figure 5). These populations were T cells (CD3^+^), T‐helper cells (CD3^+^CD4^+^), cytotoxic T cells (CD3^+^CD8^+^), Natural Killer cells (CD3^+^CD56^+^), Natural Killer‐like cells (CD3^+^CD56^+^), T‐regulatory cells (CD4^+^CD25^+^FoxP3^+^) and T‐cell receptor gamma delta‐positive cells (γδ T cells). The proportions of cells expressing cell surface markers CD11b, CD80, CD204, CCR10, and cytokines IFN‐γ, TGF‐β, IL‐23, were not altered in expression levels between the patient and control groups.

### Significant fold changes in proportions of alternative activated macrophages were observed 1–2 months post‐burn, with no relative change in monocytes or activated macrophages

The relative myeloid cell populations show no significant changes over time, or between burn patient samples and the control group samples (Supplementary figure 6). Proportions of dendritic cells (Supplementary figure 6a) were significantly reduced in the first 3 weeks post‐burn when compared to 3–18 months (*P* < 0.05). No significant changes were observed in CD11b^+^ populations, activated monocytes, or activated macrophages (Supplementary figure 6b–d). Control unstimulated activated macrophage levels (Supplementary figure 7a) favoured an M1‐like profile in control samples. The ratio of M1‐like macrophages compared to M2‐like macrophages in unstimulated burn samples was relatively similar. The stimulated activated macrophages in the control samples show 20% conversion to the M2‐like profile (Supplementary figure 7b), while the stimulated burn samples favoured an M2‐like phenotype from 1–2 months to 9–18 months post‐burn. No significant differences were detected between these ratios. Activated macrophages were assessed by fold change (FC) differences compared to control (Table 2). Alternatively activated macrophages (CD163) had significantly higher fold increases than classically activated macrophages (CD80) at 1–2 months post‐burn (*P* < 0.0012). Peripherally circulating classically activated CD86 macrophages dominated CD80 activity 1–3 weeks post‐burn (*P* < 0.0481).

**Table 2:** Alternatively activated (M2‐like) macrophages were 3.52‐fold higher than patient control levels, and significantly different from Classic (M1‐like) macrophage levels at 1–2 months post‐burn

Time post‐burn	CD80 (M1) FC	CD86 (M1) FC	CD163 (M2) FC	CD204 (M2) FC	*P*‐value
1–3 weeks	0.26	1.75	2.88	1.04	*0.0481
1–2 months	0.62	1.84	3.52	0.99	**0.0012
3–8 months	0.77	1.05	2.76	0.94	ns
9–18 months	0.74	1.30	1.96	1.25	ns

**Figure 1:**
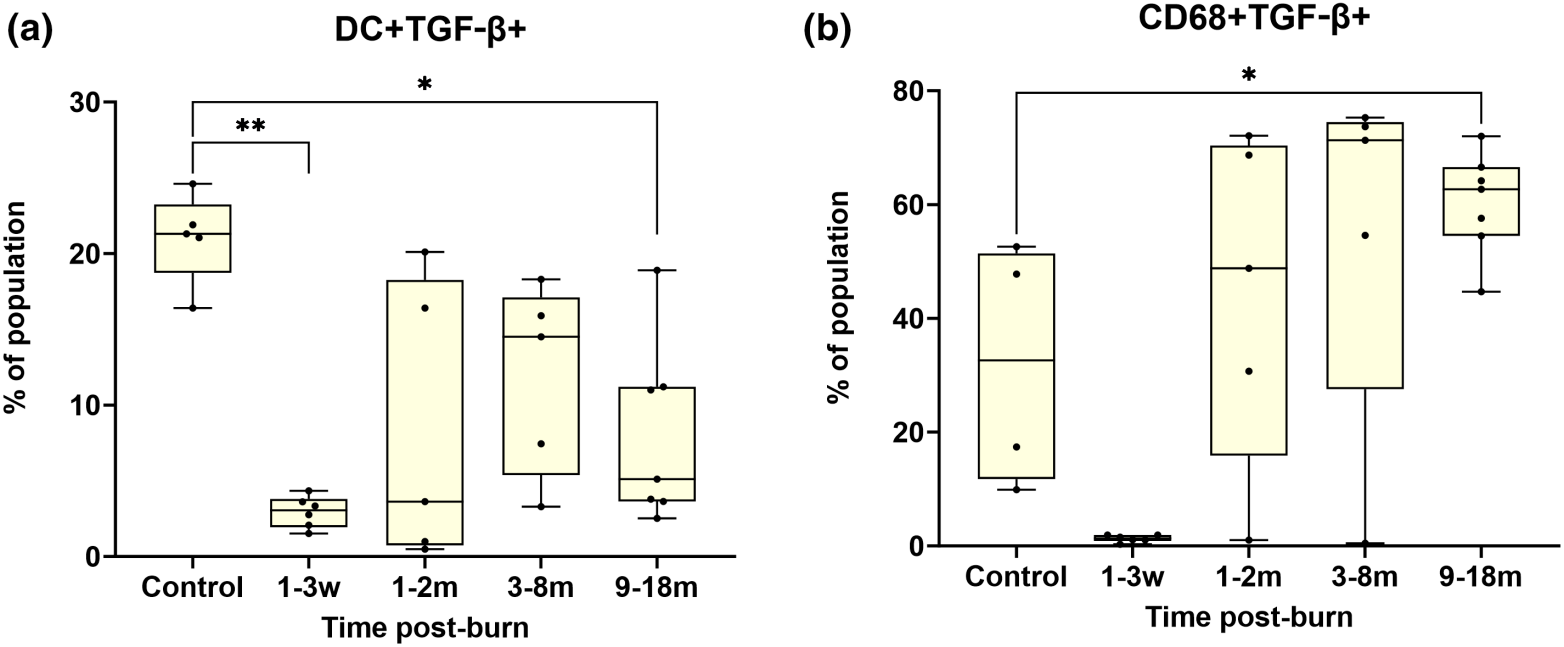
**(a)** Smaller proportions of dendritic cells (DC) expressed TGF‐β at 1–3 weeks (*P* < 0.01) and at 9–18 months post‐burn (*P* < 0.05). In contrast, more macrophages (CD68) **(b)**, expressed TGF‐β at 9–18 months post‐burn *(P* < 0.05). *P < 0.05, **P < 0.01. m, month; w, week.

Significant differences in proportional myeloid cell activity were found in the production of TGF‐β. Dendritic cells (Figure 1a) produced relatively less TGF‐β at 1–3 weeks and 9–18 months post‐burn. Circulating macrophage marker (CD68) positive cells produced more relative amounts of TGF‐β at 9–18 months post‐burn (Figure 1b).

### Burn injury is associated with changes in inflammatory and skin‐homing chemokine expression

**Figure 2:**
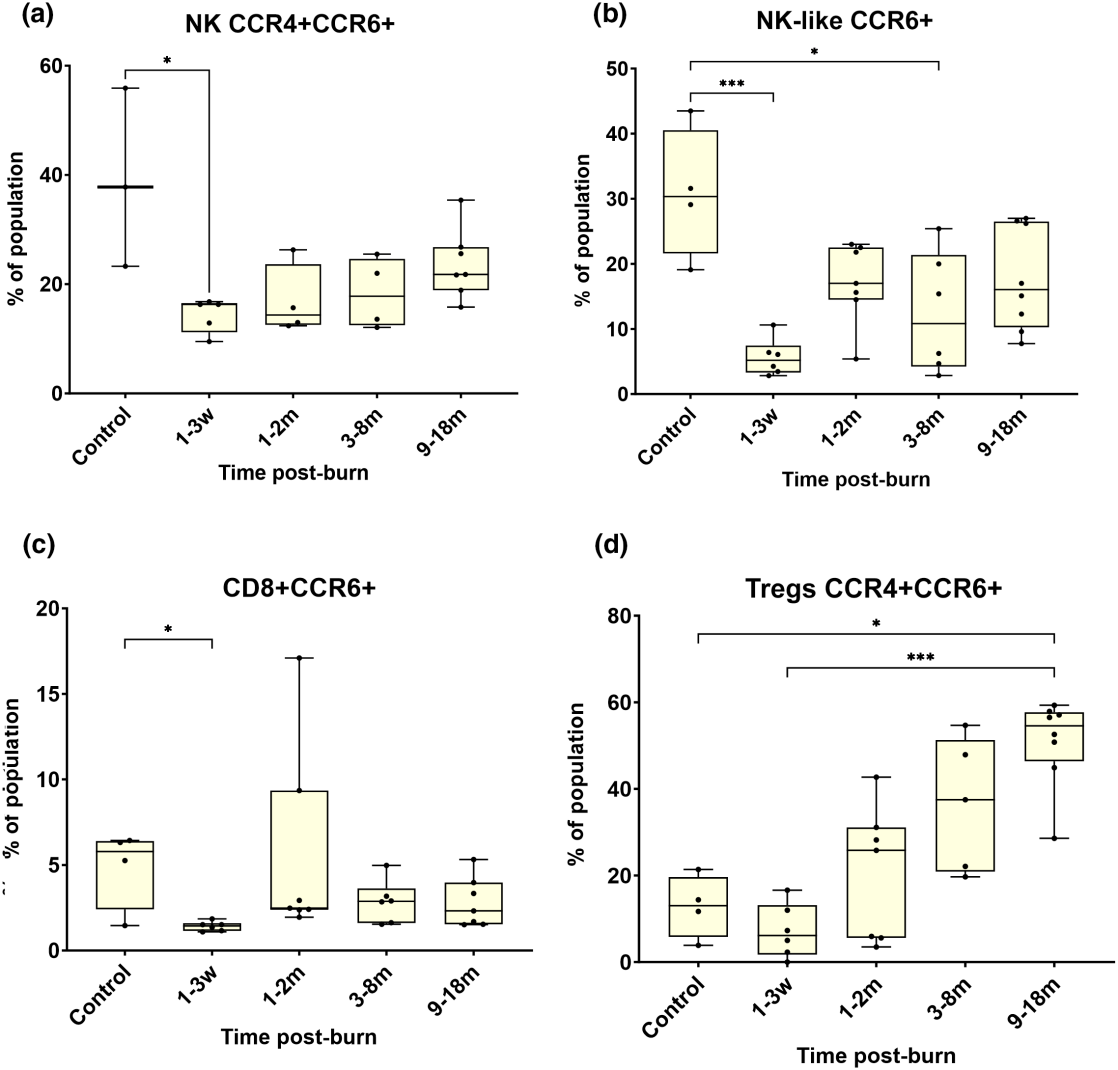
Changes to the expression levels of chemokines in Natural Killer cells, Natural Killer‐like T cells and T cells post‐burn. Double‐positive (CCR4^+^CCR6^+^) Natural Killer cells **(a)** were significantly reduced in the circulation within the first 3 weeks post‐burn. NKT‐like cells expressing CCR6^+^ **(b)** decreased in the circulation at several time points post‐burn. T‐cytotoxic CCR6^+^ cells **(c)** decreased in the first 3 weeks. Circulating levels of double‐positive (CCR4^+^CCR6^+^) T‐reg cells **(d)** were increased at 9–18 months post‐burn compared to control, and significantly increased compared to the first 3 weeks post‐burn. *P < 0.05, ***P < 0.001. m, month; w, week.

Relative expression of chemokines decreased in circulation within the first 3 weeks post‐burn, then returned to control levels in most cells, except Tregs. Statistical analysis showed that the abundance of double‐positive Natural Killer cells with CCR4 and CCR6 (Figure 2a) had proportionally reduced in circulation in the first 3 weeks post‐burn compared to healthy controls (*P* < 0.05). Natural Killer‐like T cells that were positive for CCR6^+^ were also proportionally lower than control in the first 3 weeks (Figure 2b; *P* < 0.001). Further, relative numbers of NKT‐Like^+^CCR6^+^ cells (Figure 2b) were also significantly lower than control at 3–8 months post‐burn (*P* < 0.05). Proportions of cytotoxic T cells that were positive for marker CCR6^+^ (Figure 2c), were significantly reduced compared to control in the first 3 weeks post‐burn (*P* < 0.05). Tregs with the double‐positive phenotype CCR4^+^CCC6^+^ (Figure 2d) were proportionally lower in the first 3 weeks post‐burn and were significantly elevated in circulation 18 months post‐burn (*P* < 0.001). Relative quantities of double‐positive Tregs were also significantly higher at 9–18 months post‐burn, than in controls (*P* < 0.05).

### Novel and effector T‐cell populations secrete proportionally more potent pro‐inflammatory mediators in response to burn injury

**Figure 3:**
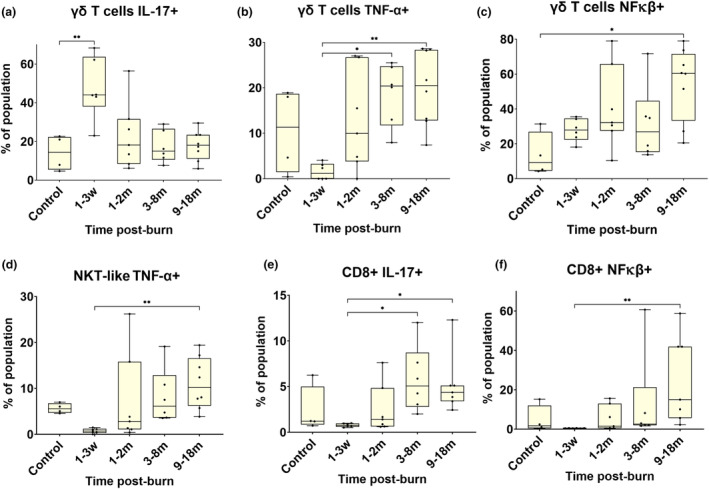
Different cell types displayed pro‐inflammatory cytokine expression. There were significantly more γδ T cells capable of producing IL‐17 **(a)**, TNF‐α **(b)** and NFκβ **(c)** post‐burn. The number of NKT‐like cells that showed increased potential for secreting TNF‐α **(d)** were significantly increased at 9–18 months post‐burn, compared to the first 3 weeks. T‐cytotoxic cells with increased expression of IL‐17 **(e)** were significantly increased in the circulation 3–18 months post‐burn, compared to the first 3 weeks. T‐cytotoxic cells expressing NFκβ **(f)** were increased at 9–18 months compared to the first 3 weeks post‐burn. *P < 0.05, **P < 0.01. m, month; w, week.

Relative elevated cytokine expression was observed in different cells at varying timepoints post‐burn as seen in Figure 3. The proportion of pro‐inflammatory γδ T cells expressing IL‐17 (Figure 3a) were increased in the first 3 weeks post‐burn compared to control (*P* < 0.01). The γδ T cells expressing TNF‐α (Figure 3b) decreased in the first 3 weeks and were significantly increased at 3–8 months (*P* < 0.05), and 9–18 months burn (*P* < 0.01). The proportion of γδ T cells positive for transcription factor NFκβ (Figure 3c), were significantly more abundant than control at 9–18 months post‐burn (*P* < 0.05). NKT‐Like cells potential for producing inflammatory cytokine TNF‐α (Figure 3d), were seen at proportionally lower levels in the first 3 weeks post‐burn and were significantly higher 9–18 months post‐burn (*P* < 0.05). Cytotoxic T cells expressing IL‐17 (Figure 3e) were relatively decreased in the first 3 weeks, which was significantly different to 3–18 months post‐burn, when the abundance was much higher (*P* < 0.01). Cytotoxic T cells expressing NFκβ (Figure 3f) were significantly higher at 9–18 months post‐burn than in the first 3 weeks post‐burn (*P* < 0.01).

### Burn injury disrupts T‐regulatory cell function, with a shift in production of anti‐inflammatory IL‐10 to inflammatory TNF‐α over time post‐burn

**Figure 4:**
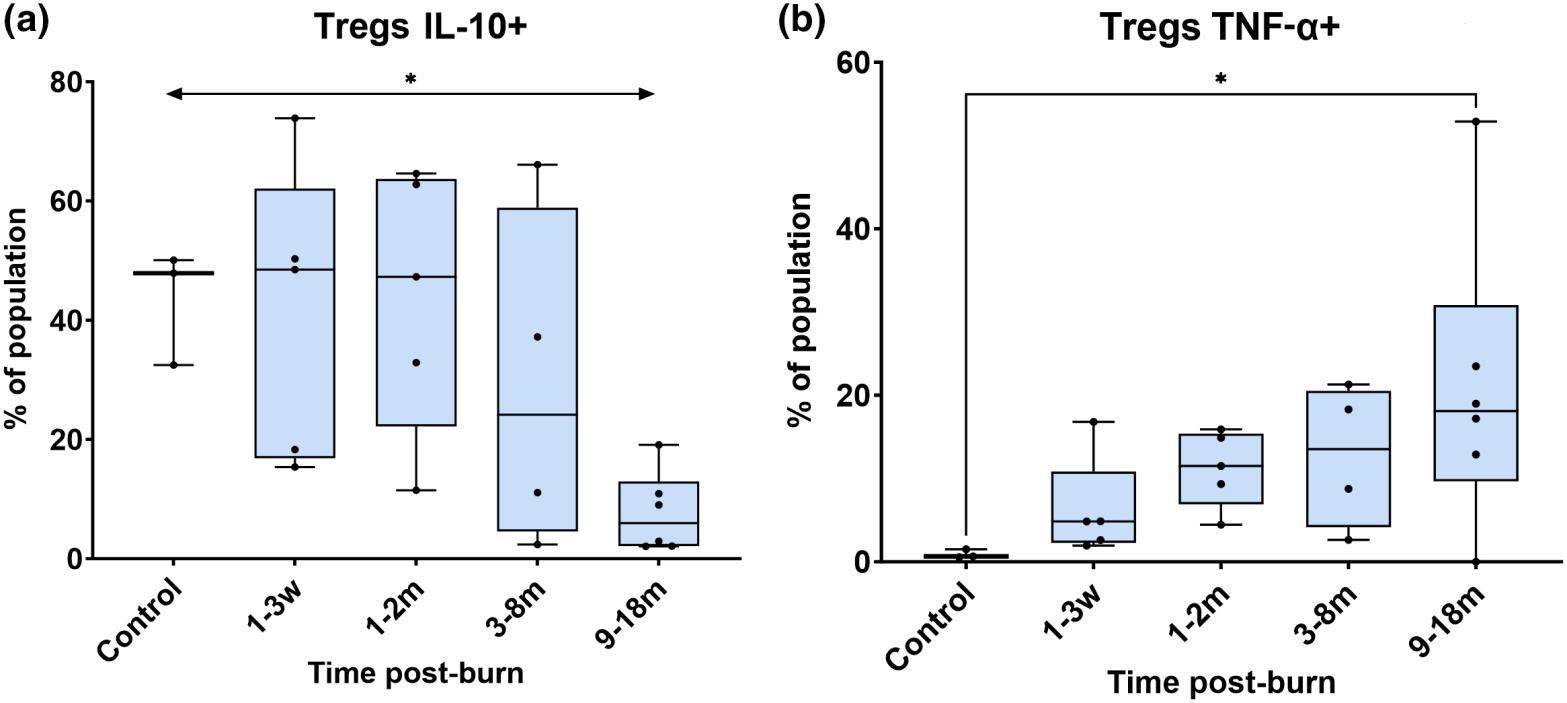
A decreased proportion of Tregs expressed IL‐10 over time post‐burn, compared to control **(a)** and there were significantly more Tregs expressing TNF‐α at 9–18 months post‐burn **(b)**. *P < 0.05. m, month; w, week.

T‐regulatory cell function changed post‐burn, as seen in Figure 4. Tregs produced relatively less IL‐10 post‐burn (Figure 4a) and increased amounts of TNF‐α (Figure 4b).

### Anti‐inflammatory actions of NKT‐like cells in early phases post‐burn injury are suggestive of an immunomodulatory function

**Figure 5:**
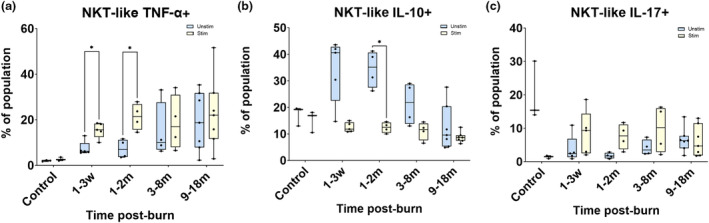
Stimulated and unstimulated NKT‐like cells had different cytokine expression. Significantly more stimulated NKT‐like cells expressed TNF‐α, for 1 week to 2 months post‐burn **(a)** (*P* < 0.05). Significant differences were seen between unstimulated and stimulated cells expressing IL‐10 at 1–2 months post‐burn (*P* < 0.05) **(b)**. No significant differences were detected between stimulated and unstimulated populations of NKT‐like cell expression of IL‐17 **(c)**. *P < 0.05. m, month; w, week.

The functional changes in NKT‐like (CD3^+^CD56^+^) cells in burn patients favoured a regulatory status while unstimulated, but became pro‐inflammatory once stimulated, as seen in Figure 5. Significantly more NKT‐like cells expressed cytokine TNF‐α on stimulation than their unstimulated / resting states (Figure 5a). In contrast, unstimulated or resting cells preferentially expressed IL‐10 (Figure 5b). No significant differences were seen in cytokine IL‐17 expression in the NKT‐like cells (Figure 5c).

### Decreased circulating levels of macrophage inflammatory Protein‐1 were observed 1–2 months post‐burn injury

Plasma samples were analysed using a LEGENDplex Human Inflammation Panel 1. Plasma levels of macrophage inflammatory protein 1 (MIP‐1) were significantly lower than control at 1–2 months post‐burn (*P* < 0.01), and returned to baseline levels at 9–18 months (*P* < 0.05; Supplementary figure 8). Three circulating cytokines failed to be recorded at detectable levels in the assay (IL‐1β, TNF‐α, IFN‐γ). The remaining detected cytokines are presented in Supplementary figure 9; however, none were significantly different to control or showed significant changes over time post‐burn.

## Discussion

This study investigated the immune profile changes in paediatric burn patients for over 18 months post‐burn. As most of the samples analysed in this study were from patients with < 10% area burns (considered non‐severe), the results suggest the immune system is responding to a burn event regardless of the burn size. Of note, the individual responses were not representative of the severity of the burns, as in several instances it was the smaller 1.5–3% area burns which were the high outliers. The results presented here also demonstrate that the response to a burn injury consists of several pro‐inflammatory markers and cytokines. In this study, the principal lymphocyte populations of T cells, macrophages and monocytes were relatively unchanged over time, as reported recently in other immune burn studies.^5^, ^6^ This challenges the longstanding burns community paradigm that burns patients are immune‐suppressed,^10^, ^18^, ^19^ and the belief that they are susceptible to infection post‐burn because of a lack of immune cells. Rather, these results suggest that burn patients aren't immuno‐suppressed but are ‘immune‐distracted’. The term ‘immune distraction’ has been recently used to describe the response to Covid‐19, and explains how previous respiratory infections could exacerbate Covid‐19 symptoms.^25^ Likewise, burn injury appears to activate a high degree of inflammatory function from existing immune cells, and is suggestive of a distracted or highly activated immune system, where some cells have altered their function in response to burn injury. For example, a population of T‐helper 1 cells expressing TNF‐α may convert to Treg‐like cells. The converted Tregs cells have the capacity to produce both cytokines based on the observed threat. This phenomenon, known as cell transdifferentiation, has also been observed in patients with colorectal cancer,^26^ oesophageal cancer,^27^ autoimmune arthritis^28^ and renal fibrosis.^29^

The expression of chemokines changed after a burn injury. The relative quantities of cells expressing chemokines CCR6 or CCR4 (NK, NKT‐like and CD8 cells) decreased in circulation within the first 3 weeks post‐burn, but then returned to their baseline levels. This indicates that they migrated to the skin and were temporarily involved in the local tissue inflammatory processes. The proportion of double‐positive (CCR4^+^CCR6^+^) Tregs initially decreased in the first 3 weeks post‐burn compared to control, and then increased 4‐fold at 18 months post‐burn. In contrast, in previous adult burn studies, double‐positive cells increased after 1 month post‐burn injury^6^ and CCR4^+^ Tregs were increased at week one post‐burn.^6^ The early activation in adults may indicate that Tregs have learned to shift from effector to memory phenotypes more efficiently. Children tend to express more effector Treg types.^30^ As individuals age, immune aging and the learned self‐antigen process allow Treg differentiation to become more efficient.^31^ Alternatively, as adult burns tend to be larger and more severe, this could also explain the early activation of Tregs.

Burn injury induces changes in effector T‐cell expression of potent pro‐inflammatory cytokines. The proportion of γδ T cells and cytotoxic T cells expressing pro‐inflammatory cytokine IL‐17 increased post‐burn, and the potential for cells to produce IL‐17 was also higher than control at multiple time points after the burn injury. The γδ T cells expressed IL‐17 in the first 3 weeks, and CD8 cells expressed IL‐17 from 3–18 months post‐burn, demonstrating that there is long‐term immune disruption after a burn injury. This is consistent with Johnson *et al*.'s work, which confirms pro‐inflammatory cytokine production is persistent for 3 years post‐burn, with children having higher levels of circulating IL‐2, IL‐7, TNF‐α and IFN‐γ.^1^ This study is the first to report increasing systemic levels of γδ T cells in paediatric burn patients. Previously, Schwacha *et al*.,^32^, ^33^, ^34^, ^35^, ^36^ confirmed the presence of these cells in a murine burn model. Mulder *et al*.^37^ more recently reported γδ T cells in humans increased in burn wound eschar from a study of adults with predominantly flame burn injuries.

The γδ T cells can be skin resident and activated by Damage‐associated molecular patterns DAMPs (e.g. S100A/HMBGB1).^33^, ^38^, ^39^ We observed a 3‐fold increase in the number of γδ T cells delivering IL‐17 into circulation in the first 3 weeks post‐burn, suggesting that they are among the first responders to a burn event. In the late phase of scar remodelling, from 9–18 months post‐burn, there are elevated levels of both TNF‐α and NFκβ, co‐expressed by γδ T cells. These two key stimulators mediate T‐cell activity by activation or elimination.^40^, ^41^ This combination of TNF‐α and NFκβ suggests the classical or canonical pathway is active after burn injury, triggering the activation of innate immunity and inflammation.^42^ In the event of NFκβ suppression and elevated TNF‐α, this is indicative of the AICD (activation‐induced cell death) pathway and T‐cell apoptosis, allowing overactive T cells to be eliminated.^43^, ^44^ When the AICD pathway is defective, the overactive T cells remain in circulation, leading to auto‐reactivity and autoimmunity in murine models.^45^ The aforementioned classical pathway in this late phase of burn injury suggests long‐term immune activation and inflammation is occurring.

NKT‐like cells are known to play roles in response to viruses or infections, tumor microenvironments and onco‐immunology.^46^, ^47^, ^48^ NKT‐like cells are also previously undocumented in burns. Here for the first time, we report that NKT‐like cells appeared to be one of the first responders to a burn event, with a significant decrease in circulation 1–3 weeks post‐burn. Notably, these cells appear to be immunomodulatory in nature post‐burn, with a reduced proportion producing TNF‐α and an increased proportion producing IL‐10. NKT‐like cells have been previously reported by Wang *et al*.^49^ to secrete the immune suppressive transcription factor FoxP3 in the regulatory pathway. The suppressive factors being produced by NKT‐like cells in this study are suggestive of a protective capacity, while their original function remains between the transition from innate and adaptive, here they appear immune‐regulatory in nature.

The proportions of monocytes and macrophages were largely unchanged post‐burn, but the unstimulated control samples demonstrated a classical M1‐like profile, while the stimulated burns patient samples favoured an alternative M2‐like profile. Activated macrophages from this study were most abundant at 1–2 months post‐burn. Within this timeframe, M1‐like macrophages were 1.84‐fold higher than control, and M2‐like macrophages were 3.52‐fold higher than control (Table 2). Macrophage hyperactivity in the early burn phase is associated with fibrosis and scarring, and an early increase in CD86 (M1‐like marker) has been linked to burn hypertrophic scarring.^50^, ^51^ M1‐like and M2‐like macrophages use different metabolic processes. M1‐like macrophages use glycolysis for metabolism, while M2‐like use mitochondrial oxidative phosphorylation as energy.^52^ When M1‐like macrophages become hyperactive, they release excess metabolic byproducts that can switch their phenotype to M2‐like polarisation.^53^ This has been observed in a murine model,^54^ where macrophage hyperactivity post‐burn is independent of burn size. Removing available glucose for M1‐like macrophages to use and increasing the insulin sensitivity with metformin, switches the phenotype to M2‐like.^55^ Insulin also promotes the phenotype switch of M1‐like to M2‐like in diabetic rat models.^56^ A greater proportion of macrophages expressing TGF‐β were seen post‐burn (Figure 1). Macrophage‐secreted TGF‐β contributes to fibroblast activation, which causes scarring and contractures.^57^, ^58^ Unsurprisingly, all of the burn patients in this study had measured scar thickness of more than 3 mm, all showed delayed healing, and all had repeated burn procedures; these are all predictors of hypertrophic scarring.^59^, ^60^ MIP‐1 is a macrophage and monocyte chemoattractant. It has been shown that lower levels can reduce angiogenesis, macrophage recruitment, and contribute to delayed healing.^61^ In this study, the levels of MIP‐1 in the plasma of patients were significantly lower than in the control group at 1–2 months post‐burn. These patients had delayed healing, which indicates that MIP‐1 plays a critical role in both macrophage recruitment and wound healing.

**Figure 6:**
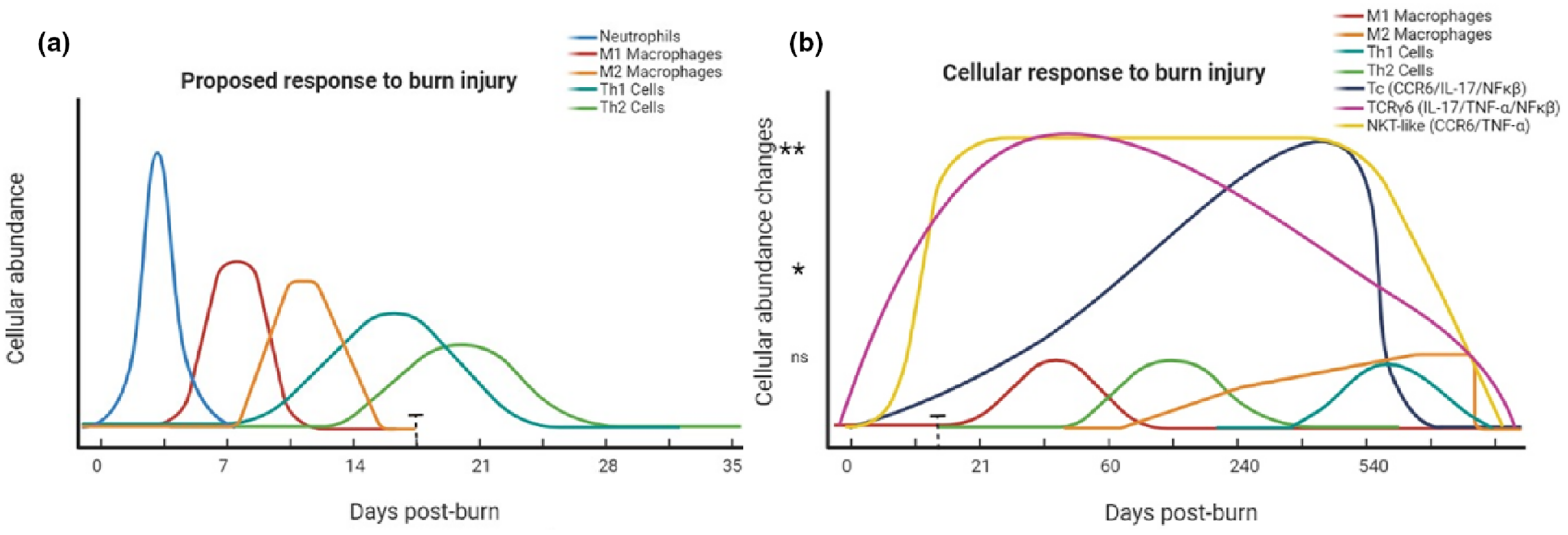
**(a)** Proposed immune cell responses to a burn injury, based on published literature.^6^, ^62^, ^63^, ^65^, ^72^, ^73^ Inflammation dominates the first 2 weeks post‐burn, and if the environment is favorable, wound healing should occur before 21 days post burn. Actual immune changes seen in this study in burns patients over time **(b).** *P < 0.05, **P < 0.01, ns, not significant.

The expected trajectory of cell behaviour during wound healing, particularly in burns and wound reviews, is well‐documented.^62^, ^63^, ^64^, ^65^ Typically, immune cells are reported to peak at specific times and then return to baseline levels before or on the 21st day post‐burn injury. Instead of the inflammatory response by immune cells (Th1/M1‐like) being mitigated by the regulatory side (Th2/Treg/M2‐like; Figure 6a), the present findings found that these principal lymphocyte groups were not significant in presentation after burn injury. Instead, highly specialised cells such as γδ T cells and NKT‐like cells mediated the post‐burn response (Figure 5b). These specialised types of cells are usually reported in patients with high viral loads and cancer.^46^, ^66^, ^67^ Additionally, we found here that immune dysregulation occurs for 18 months post‐burn and does not appear to resolve, which is also contrary to the classic wound healing response. Pro‐inflammatory mediators (Th17, TNF‐α, NFκβ, CCR6) remained in circulation at 18 months post‐burn. The predicted ‘tissue‐resolving’ response (Th2, Tregs, IL‐10, M2‐like) was not significant, or not reflective or equivalent to the inflammatory response. T‐regulatory cells are usually regulators of immune homeostasis, but in this study, they displayed an inflammatory profile. Proportions of T‐regulatory cells in this study produced less ‘anti‐inflammatory’ IL‐10 over time, and more ‘pro‐inflammatory’ TNF‐α, and expressed inflammatory and skin‐homing markers for up to 18 months post‐burn injury. Furthermore, in this study there were several cell populations which had cytokine expression profiles different to their natural states, in response to burn injury. This may suggest that classifications of immune cell populations as strictly inflammatory or suppressive, in the context of a burn injury, may be an oversimplification.^68^ This study has highlighted that there is a lasting pro‐inflammatory immune profile for paediatric burn patients with post‐burn scarring. Future research should investigate methods to resolve this inflammatory state, to improve burn patient outcomes or reduce future co‐morbidities for burn patients.

There are several limitations in this study that need to be considered. Firstly, apart from one patient, all the burn patients had acquired nosocomial infections during their treatment period and were subsequently given antibiotics. This means that the immune cell and cytokine responses observed in these patients may have been affected by the infection or the use of antibiotics. Secondly, the sample size was limited, as there were only six burn patients with multiple blood collections in the timeframe of the study. While this is a positive outcome for the patients, more burn patients with multiple longitudinal sample collections would have added to the breadth of data presented. Additionally, many paediatric burn samples are collected only on the days when patients required treatment, and the timing for these collections varied depending on patient or burn‐specific reasons. Therefore, the sample timepoints varied, and grouping them may have obscured some of the increases or decreases in marker analysis, and some fluctuations in populations may have been missed. Moreover, the patients within this study had all returned for repeated procedures, indicating they were more severely affected by their burns and this is not representative of all paediatric burn patients.^69^ Therefore, their observed immune response may be greater than for typical paediatric burn patients. The cryopreservation and subsequent thawing of cells presents a limitation, cells may undergo some intracellular damage through this process, such as membrane leakage causing the cells to be osmotically stressed.^70^ However, the PBMCs in this study were frozen quickly, using vitrification, which in recent years is the gold standard in cryopreservation as it typically yields high recovery rates of cells.^71^

This study demonstrates long‐term cellular immune changes in paediatric burn patients after a burn injury. This study also identified some novel cell populations involved in recruitment and inflammation post‐burn, such as NKT‐like and γδ T cells. We also saw that the principal lymphocyte populations did not increase or decrease in number post‐burn but increased in capacity and function. The results reported here reflect other post‐burn immune cell studies. The increase in cell specificity and effector cells is a common finding in cell studies post‐burn injury.^6^ The increase and sustained transition to effector and pro‐inflammatory cell profiles contributes to post‐burn systemic inflammation and immune dysregulation.^5^ Significant immune profile changes in children have been reported at 3 years post‐burn, with increased expression of inflammatory surface proteins on immune cells, and decreased Diphtheria, Tentaus and Pertussis (DPTa) vaccine titers.^5^ These results all suggest a sustained immunological change in burn survivors, and the mediators of this response may be potential targets for novel burn treatments, to improve healing time and reduce scarring. It remains unclear why the regulatory side of the immune system is not equal in response to the inflammatory response after healing, and why this systemic inflammation is sustained for 18 months post‐burn. Future research would benefit from including comparisons of patients with optimal and poor healing outcomes, relative to their immune response.

## Methods

### Patient recruitment and ethics approvals

This study involved the recruitment of paediatric burn patients who presented with a burn injury at the Queensland Children's Hospital, Brisbane. Ethical approval was obtained for the collection of whole blood samples (HREC/19/QCHQ/48683, SSA/19/QCHQ/48683, QUT#2021000233). Samples were de‐identified before processing in the lab. Clinical data concerning the patient demographics, burn severity and healing outcomes were obtained from patient medical records. The children and parents provided informed consent and assent where possible, when recruited to the study and were able to withdraw from the study at any time.

### Collection and isolation of peripheral blood mononuclear cells

Whole blood samples were collected in Becton Dickson vacutainers containing lithium heparin (Becton Dickson, New Jersey, USA), and processed within 24 h. Centrifugation was used to isolate the plasma, plasma was then frozen in protein Lo‐bind tubes (Sigma‐Aldrich, St. Louis, USA) at −80°C and stored for future analysis. Peripheral blood mononuclear cells were isolated by density gradient centrifugation in Lymphoprep (Stemcell Technologies, Vancouver, Canada), frozen down with RPMI media, 2% Fetal Bovine Serum (GIBCO, Thermofisher, Paisley, UK) and 15% Di‐Methyl Sulphoxide (Sigma‐Aldrich), and stored in liquid nitrogen at −196°C for future analysis.

### PBMC stimulation

Frozen PBMCs were thawed in RPMI containing 20% FBS and washed twice in RPMI + 10% FBS. PBMC were stimulated *in vitro* to reflect inflammatory conditions and encourage functional and cytokine responses. The cells were resuspended in RPMI + 10% FBS at 1 × 10^6^ cells mL^−1^ and stimulated for 4 h with 100 ng mL^−1^ of Phorbol myristate acetate (PMA, Stemcell Technologies) and 1 μg mL^−1^ Ionomycin (Stemcell Technologies). To enhance intracellular cytokine detection, 1 μg of Golgi‐Stop (BD Biosciences, New Jersey, USA) was added. Stimulations were performed in cell culture incubators at 37°C. Cells were harvested, washed and resuspended in Dulbecco's phosphate buffered saline (GIBCO, Thermofisher). A schematic for this process can be found in Supplementary figure 1.

### PBMC staining

Three flow cytometry panels were designed to identify 25 cell markers, chemokines and cytokines, as shown in Supplementary table 1. After stimulation, the PBMCs were stained with FVS700 (BD Biosciences), for live/dead identification, were stained with extra‐cellular markers, fixed, permeabilised, blocked and then stained for intracellular markers. The PBMCs were stained with fluorescent antibodies (BD Biosciences, R&D systems, Minneapolis, USA, Thermofisher) as described in Supplementary table 1, to ascertain cellular lineage, phenotypes, function and functional potential. To avoid channel crosstalk, spill over between channels and overuse of antibodies, titration experiments and compensation calculations were performed before each run for accurate inter‐run results, and initially performed for all antibodies (Supplementary table 2). Panel 1 (Supplementary figure 2) shows the abundance of effector and helper cells, NK, NKT, cytotoxic T cells, Th17, Th1, Th2, Th22, Tregs, γδ T cells and related functional chemokines and cytokines. Panel 2 (Supplementary figure 3) shows the abundance of dendritic cells, monocytes, naïve macrophages, macrophages in the M1 phenotype: CD80, CD86 and macrophages of M2 phenotype: CD163, CD204, and related functional chemokines, and cytokines. Panel 3 (Supplementary figure 4) shows the T‐regulatory cell population, and related functional chemokines and cytokines. All antibody mixes were diluted with FACS buffer, and all washes were performed with FACS buffer (AutoMACS, Miltenyi, Germany). Cells were stained with extra‐cellular antibodies on ice for 45 min. Cells were fixed/permeabilised with Fix/Perm buffer, Diluent buffer and Perm/Wash Buffer (BD Pharmingen™, New Jersey, USA). Following fixing, intracellular staining was also performed on ice for 45 min with intracellular antibodies listed in Supplementary table 1. Cells were washed and resuspended in AutoMACS buffer prior to Flow Cytometry Analysis. A single healthy control sample was used as an internal control for each experiment and was stained as per protocol.

### Flow cytometry

FACS data acquisition was performed on a CytoFLEX S flow cytometer, (Beckman Coulter, Minnesota, USA), then analysed with the software program CytExpert (Beckman Coulter). Populations were manually gated on FlowJo (version 10.8.1, BD, USA). Light scatter parameters, FSC‐A and SSC‐A were used to identify cells based on size and to identify target cells. Doublet cells were removed by FSC. From this population, live cells were gated based on negative Live/Dead staining. Gating strategies were developed for the three different panels which were designed to reflect both pro‐inflammatory and anti‐inflammatory immune profiles (Supplementary figures 2–4).

### Cytokine analysis by multiplex cytokine profiling by the flow cytometry assay kit ‐ the Human Inflammation panel

Plasma was isolated from whole blood samples and analysed using the LEGENDplex Human Inflammation Panel 1, #740809, Lot No B356588 (BioLegend, San Diego, USA), following the manufacturer's instructions. Thirteen cytokines were measured: IL‐1β, IFN‐α2, IFN‐γ, TNF‐α, MCP‐1, IL‐6, IL‐8, IL‐10, IL12p70, IL‐17A, IL‐18, IL‐23 and IL‐33. Reagents, buffers and standards were prepared for the panel as per the manufacturer's instructions. Plasma samples were thawed and diluted with LEGENDplex assay buffer at 1:2 as required so they were within the expected ranges of the standard curve. Data acquisition was performed on Cytoflex S (Beckman Coulter) using the manufacturer's instructions for gain and gating parameters. FACS data was uploaded to BioLegend Qognit software analysis browser (Version 2023‐02‐15@2019 Qognit, Inc., San Carlos, USA). Sample concentrations were extrapolated from the standard curve, as instructed by the manufacturer.

### Statistics

PBMC populations were manually gated on FlowJo v10.2 software program (FlowJo, LLC, Ashland, USA) and data was exported to GraphPad PRISM software for analysis (GraphPad software LCC, version 9.5.1.733, San Diego, USA). Both the PBMC and LEGENDplex data were assessed for differences between healthy controls and each burn time interval, and between different post‐burn timepoints using a Kruskal–Wallis ANOVA.

## Author contributions

**Donna Langley:** Conceptualization; data curation; formal analysis; funding acquisition; investigation; methodology; project administration; resources; software; validation; visualization; writing – original draft; writing – review and editing. **Kate Zimmermann:** Methodology; writing – review and editing. **Emma Krenske:** Writing – review and editing. **Giorgio Stefanutti:** Resources. **Roy M Kimble:** Resources; writing – review and editing. **Andrew JA Holland:** Writing – review and editing. **Mark W Fear:** Writing – review and editing. **Fiona M Wood:** Writing – review and editing. **Tony Kenna:** Conceptualization; investigation; methodology; supervision; visualization; writing – original draft. **Leila Cuttle:** Funding acquisition; investigation; resources; supervision; visualization; writing – original draft.

## Conflict of interest

The authors have no conflicts of interest to declare.

## Supporting information


Supporting Information
