# Supplementary material for: Unremitting pro‐inflammatory T‐cell phenotypes, and macrophage activity, following paediatric burn injury
Source: Clin Transl Immunology. 2024 Mar 8;13(3):e1496. doi: 10.1002/cti2.1496 (PMC10921233; doi:10.1002/cti2.1496)
Supplement: Supplementary file 1 — Supporting Information [file CTI2-13-e1496-s001.docx]

**SUPPLEMENTARY DATA**

**Supplementary figure 1.** Schematic of flow cytometry PBMC sample preparation. The cells were stimulated for 4 hours and the whole flow cytometry protocol was performed in one day.

**Supplementary table 1.** Markers and panels used for flow cytometry. BD – Bioscience, E – Extra-cellular marker, I – Intra-cellular marker, L – Lineage, D – Dump channel, Mφ – Monocyte, M1 – M1-like macrophage, M2 – M2-like macrophage, Th- T-helper cell, Treg – T-regulatory cell.

|  | Marker | Fluorophore | Clone | Supplier | Cytoflex Bandpass | Intra/  Extra | Titration | Panel 1 | Panel 2 | Panel 3 |
| --- | --- | --- | --- | --- | --- | --- | --- | --- | --- | --- |
|  | Live/dead | FVS | 700 | BD |  |  |  |  |  |  |
| 1 | CD3 | APC-H7 | SK7 | BD | R780/60 | E | 1920 | L | - | L |
| 2 | CD4 | BV605 | RPA-T4 | BD | V610/20 | E | 480 | L | - | L |
| 3 | CD8 | BV650 | RPA-T8 | BD | V660/20 | E | 960 | L | - | D |
| 4 | CD11b | BV650 | D12 | BD | V660/20 | E | 1920 | - | Mφ | - |
| 5 | CD11c | PE-Cy7 | B-LY6 | BD | Y780/60 | E | 960 | - | DC | - |
| 6 | CD14 | BV480 | MPhiP9 | BD | V525/40 | E | 240 | - | M | - |
| 7 | CD25 | PE-Cy5 | M-A251 | BD | Y690/50 | E | 1920 | L | - | L |
| 8 | CD56 | BB700 | NCAM16.2 | BD | B690/50 | E | 240 | L | - | Dump |
| 9 | CD68 | PE-CF594 | Y1/82A | BD | Y610/20 | E | 240 | - | Mφ |  |
| 10 | CD80 | APC-H7 | L307.4 | BD | R780/60 | E | 60 | - | M1 |  |
| 11 | CD86 | PE-Cy5 | IT2.2 | BD | Y690/50 | E | 960 | - | M1 |  |
| 12 | CD163 | BV421 | GHI/61 | BD | V450/45 | E | 240 | - | M2 |  |
| 13 | CD194 (CCR4) | PE-Cy7 | 1G1 | BD | Y780/60 | E | 60 | Th | - | Th |
| 14 | CD196 (CCR6) | PE-CF594 | 11A9 | BD | Y610/20 | E | 960 | Th | - | Th |
| 15 | CD204 | BB700 | U23-56 | BD | B690/50 | E | 960 | - | M2 | - |
| 16 | CCR10 | PE | 1B5 | BD | Y585/42 | E | 960 | Th22 | - | - |
| 17 | IFN-γ | APC | 4S.B3 | BD | R660/20 | I | 480 | - | M1 | Th1 |
| 18 | IL-17A | BV421 | N49-653 | BD | V450/45 | I | 480 | Th17 | - | - |
| 19 | IL-23 | PE | C8.6 | Thermofisher | Y585/42 | I | 3840 | - | M2 | - |
| 20 | NF-κβ | PE-CY7 | p65 (Ps529) | BD | Y780/60 | I | 960 | Th | - | - |
| 21 | TCRγδ | BV480 | B1 | BD | V525/40 | E | 960 | Th | - | - |
| 22 | TGF-β | AF488 | TW4-9E7 | BD | B525/46 | I | 480 | - | M2 | - |
| 23 | TNF-α | AF488 | MAb11 | BD | B525/46 | I | 480 | Th1,Th22 | - | - |
| 24 | FOX-p3 | PE | 259D/C7 | BD | Y585/42 | I | 960 | - | - | Tregs |
| 25 | IL-10 | BV421 | JES3-9D7 | BD | V450/45 | I | 240 | - | - | Tregs,Th2 |

**Supplementary table 2.** Titration experiments performed for every antibody, below is one full example of CD3. The 08 titration was chosen because it has the greatest separation of positive and negative cells.

|  | Time/Cells/Single Cells/Live cells/CD3 +ve \| Mean (Comp-FL5-A :: R780-60-A) | Time/Cells/Single Cells/Live cells/CD3 -ve \| Mean (Comp-FL5-A :: R780-60-A) | Time/Cells/Single Cells/Live cells/CD3 -ve \| Robust SD (Comp-FL5-A :: R780-60-A) | Formula ((<Cell column="Time/Cells/Single Cells/Live cells/CD3 +ve \| Mean (Comp-FL5-A :: R780-60-A)" relativeRow="0" />-<Cell column="Time/Cells/Single Cells/Live cells/CD3 -ve \| Mean (Comp-FL5-A :: R780-60-A)" relativeRow="0" />)/(2*<Cell column="Time/Cells/Single Cells/Live cells/CD3 -ve \| Robust SD (Comp-FL5-A :: R780-60-A)" relativeRow="0" />)) |
| --- | --- | --- | --- | --- |
| 01 Unstained.fcs | n/a | 23.3 | 205 | * |
| 02 CD3 APC-H7 1.3840.fcs | 3317 | 203 | 301 | 5.17 |
| 03 CD3 APC-H7 1.1920.fcs | 6372 | 328 | 397 | 7.61 |
| 04 CD3 APC-H7 1.960.fcs | 11685 | 533 | 584 | 9.55 |
| 05 CD3 APC-H7 1.480.fcs | 21436 | 743 | 743 | 13.9 |
| 06 CD3 APC-H7 1.240.fcs | 35126 | 996 | 940 | 18.2 |
| 07 CD3 APC-H7 1.120.fcs | 55391 | 1258 | 1151 | 23.5 |
| 08 CD3 APC-H7 1.60.fcs | 73479 | 1547 | 1387 | 25.9 |
| 09 CD3 APC-H7 1.30.fcs | 83717 | 1885 | 1703 | 24 |


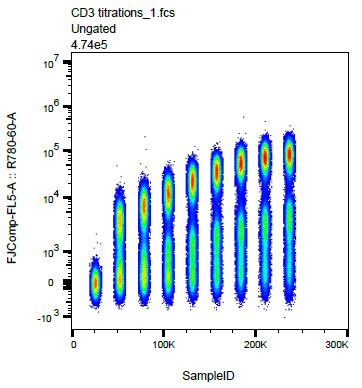


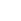


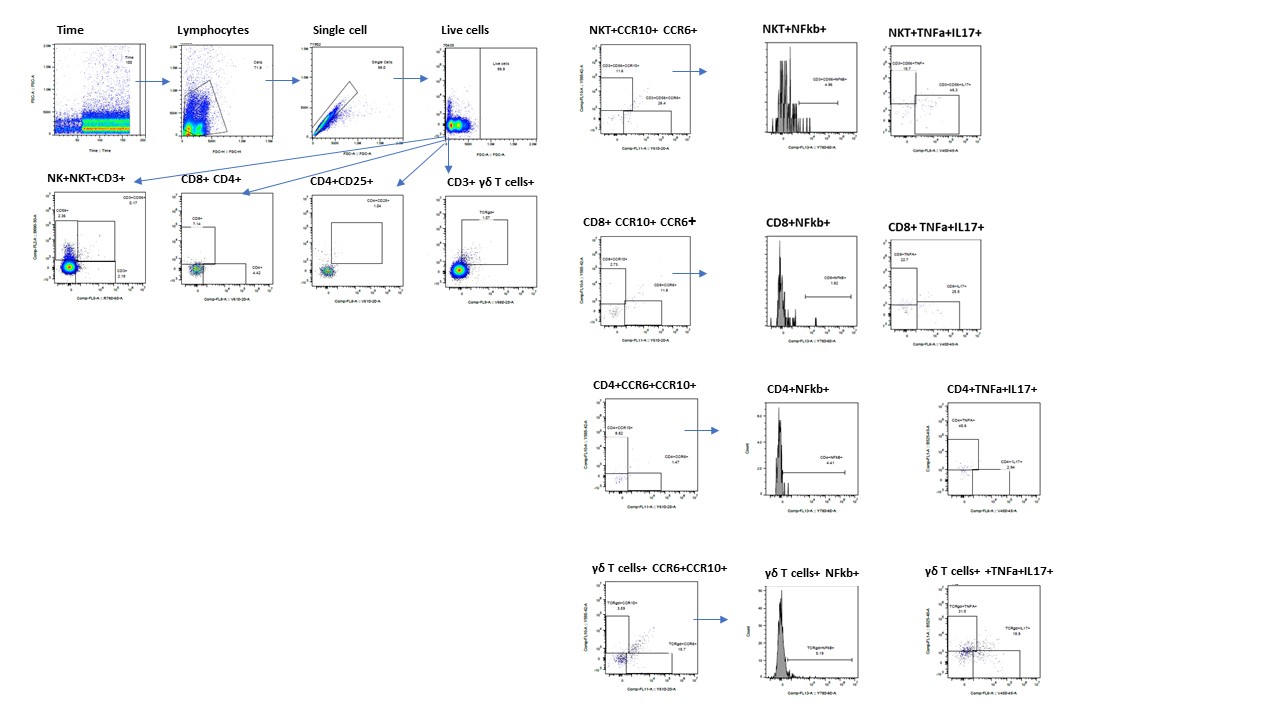


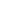

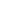

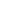

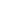

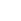


**Supplementary figure 2.** The gating strategy used to quantify the abundance of T-cell subsets in Panel 1.


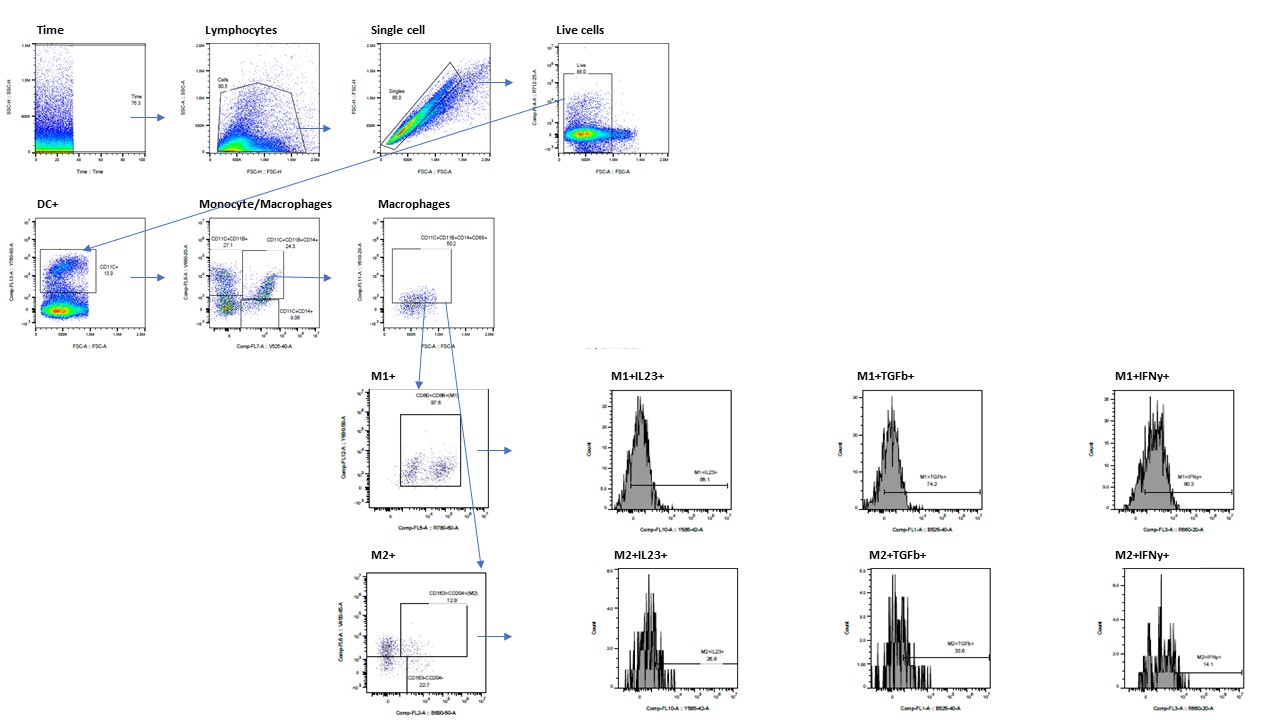


**Supplementary figure 3:** The gating strategy used to quantify the abundance of M1 and M2 Macrophages in Panel 2.

**
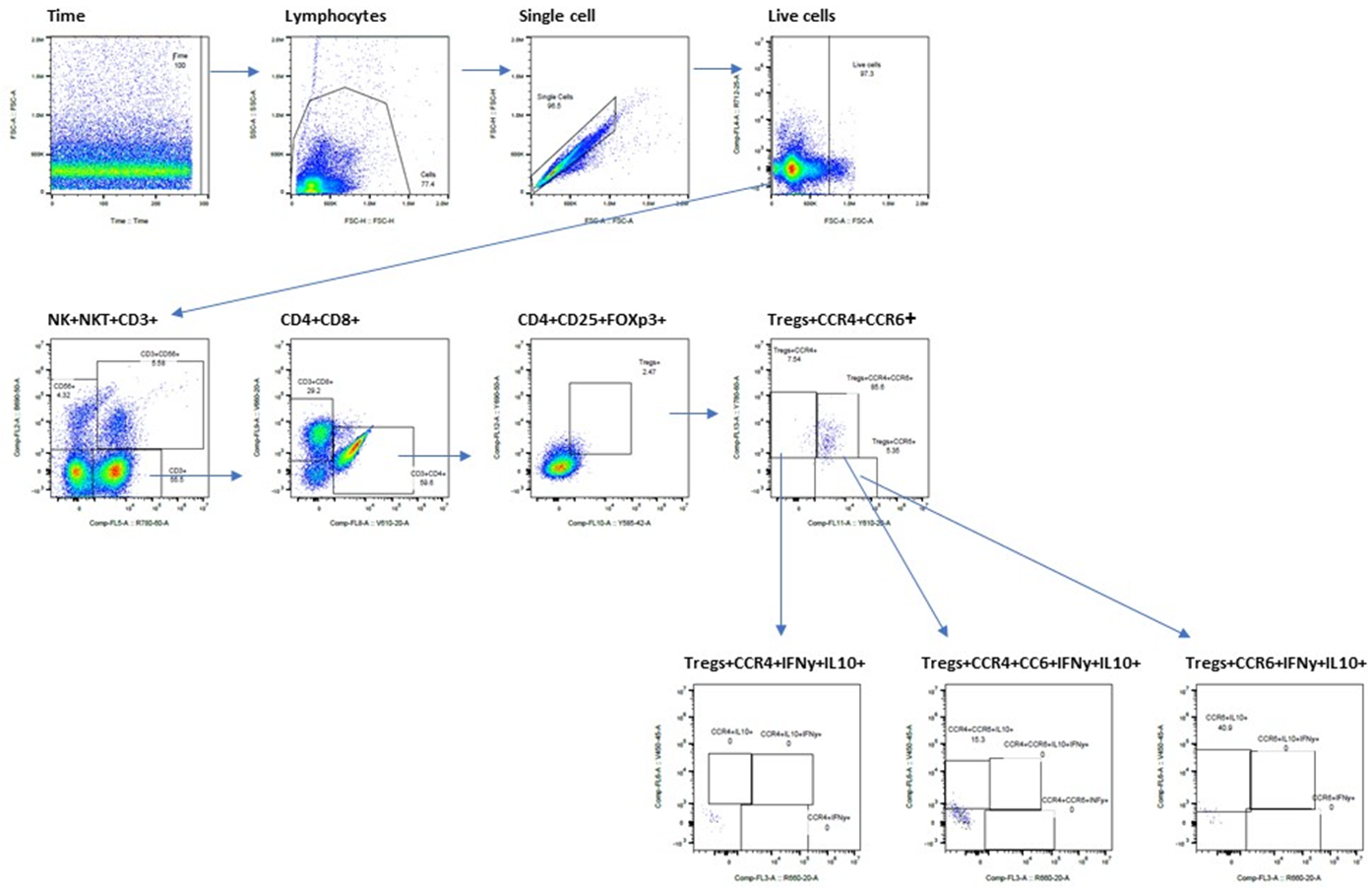
**

**Supplementary figure 4:** The gating strategy used to quantify the abundance of T regulatory cells in Panel 3.

**
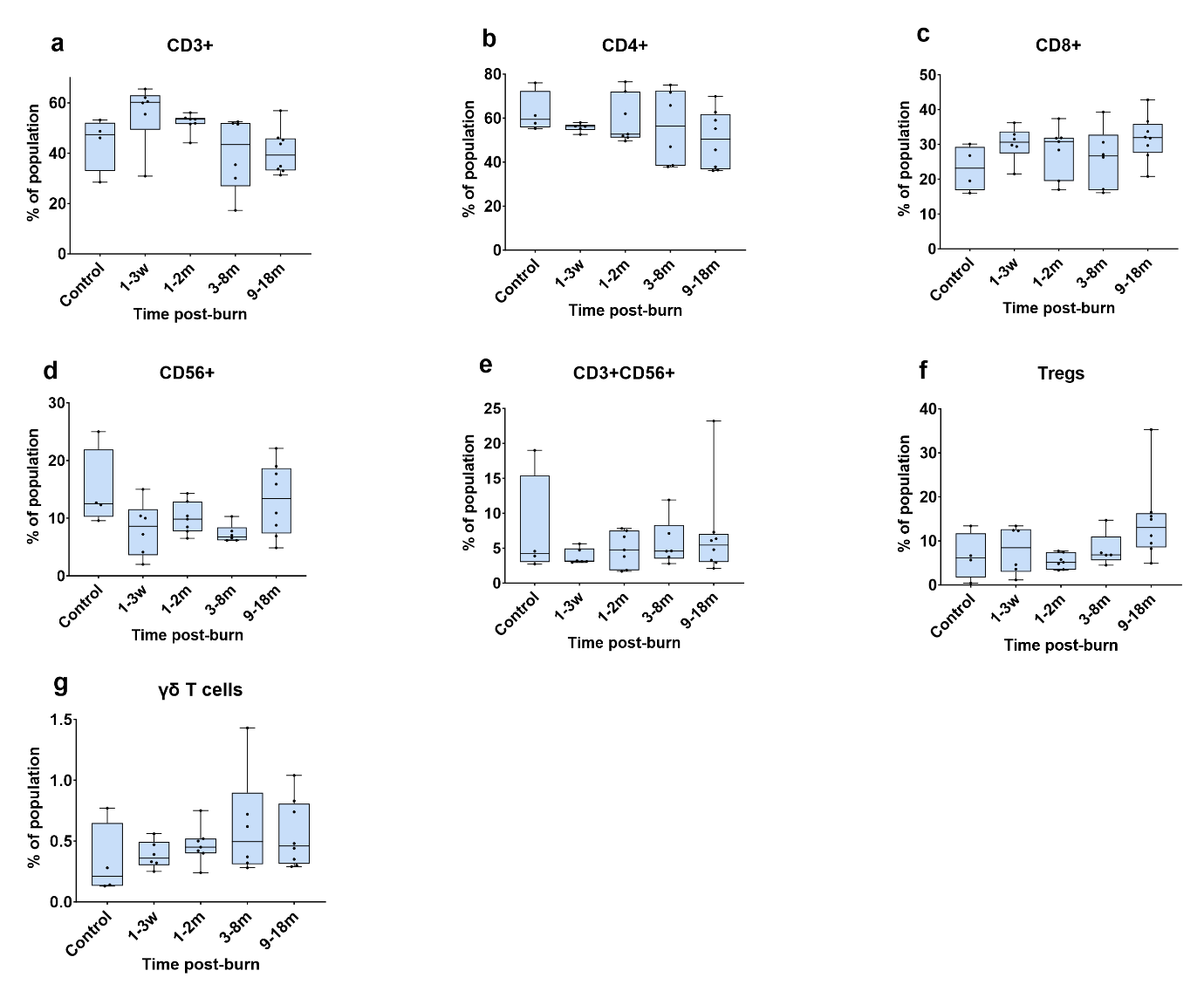
**

**Supplementary figure 5.** Proportions of principal lymphocyte populations showed no significant differences relative to control; (a) naïve T cells, (b) T-helper cells, (c) T-cytotoxic cells, (d) Natural Killer cells, (e) NKT-like cells, (f) T-Regulatory, (g) γδ T cells. w, week; m, month.


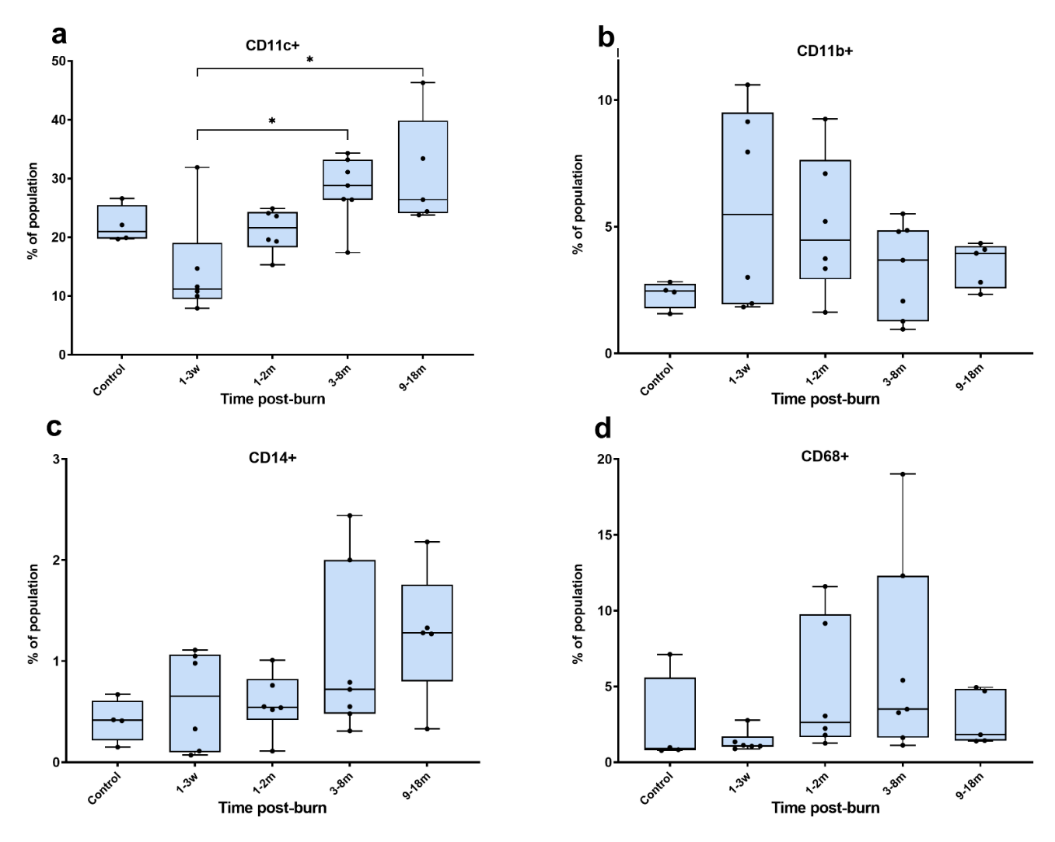


**Supplementary figure 6.** Proportions of dendritic cells **(a)** are significantly higher in circulation 3-18 months post-burn compared to the first 3 weeks (*P* < 0.05). No significant changes are seen post-burn in CD11b^+^ **(b)**, activated Monocytes **(c),** or activated Macrophages **(d)**. w, week; m, month.


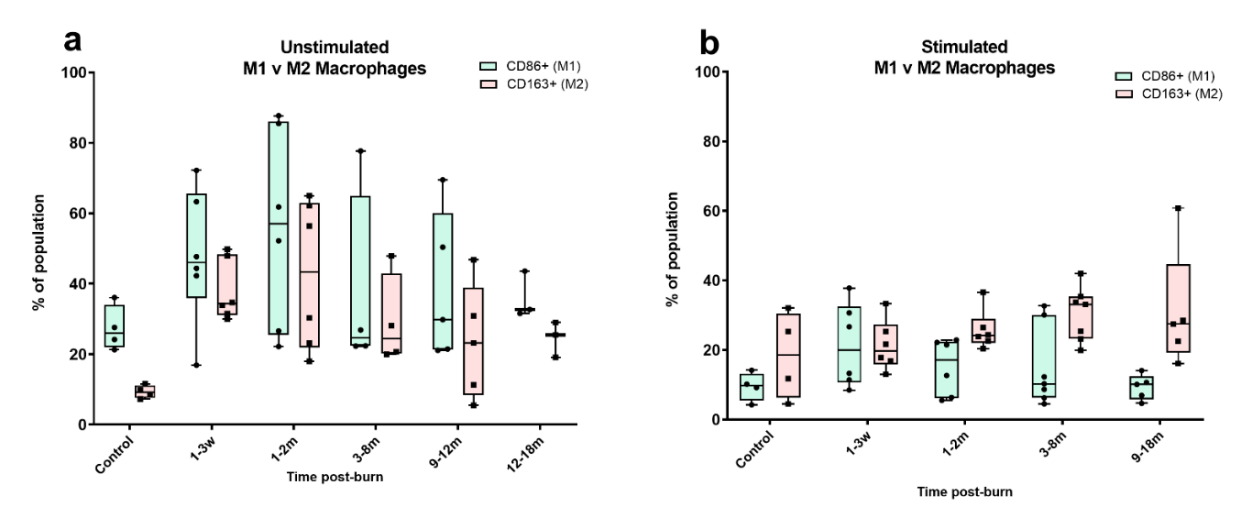


**Supplementary figure 7.** Proportions of unstimulated activated macrophages **(a)** favoured an M1-like profile in control samples, with the post-burn M1 / M2 ratio relatively similar. The stimulated macrophages **(b)** in control samples show 20% conversion to the M2-like profile, while the burns timepoints favour an M2-like phenotype from 1–2 months post-burn onward. There were no significant differences detected for these ratios. w, week; m, month.


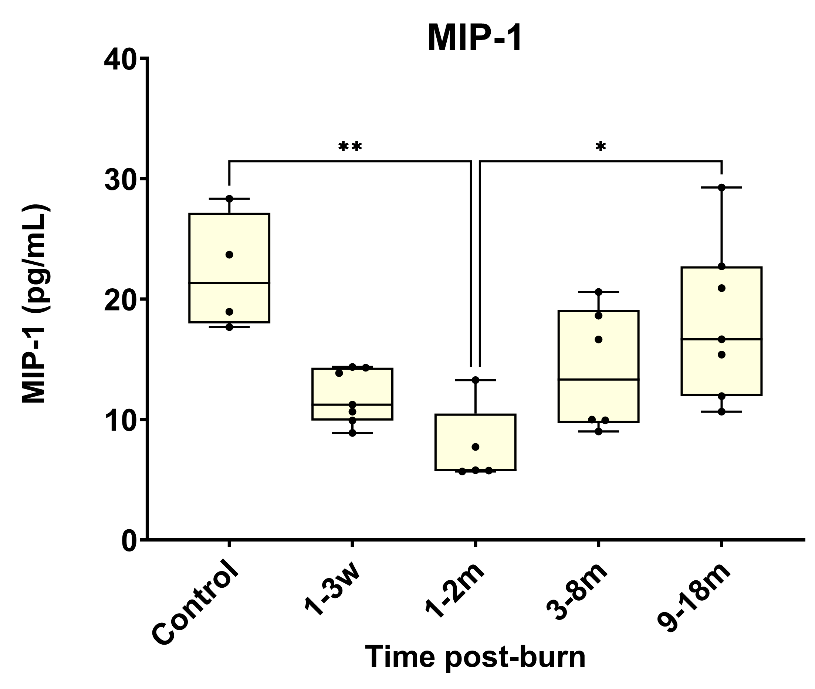


**Supplementary figure 8.** Plasma levels of MIP-1α were significantly lower than control group at 1–2 months post-burn (*P* < 0.01) and 9–18 months (*P* < 0.05), plasma results from LEGENDplex Human Inflammation Panel 1. w, week; m, month.


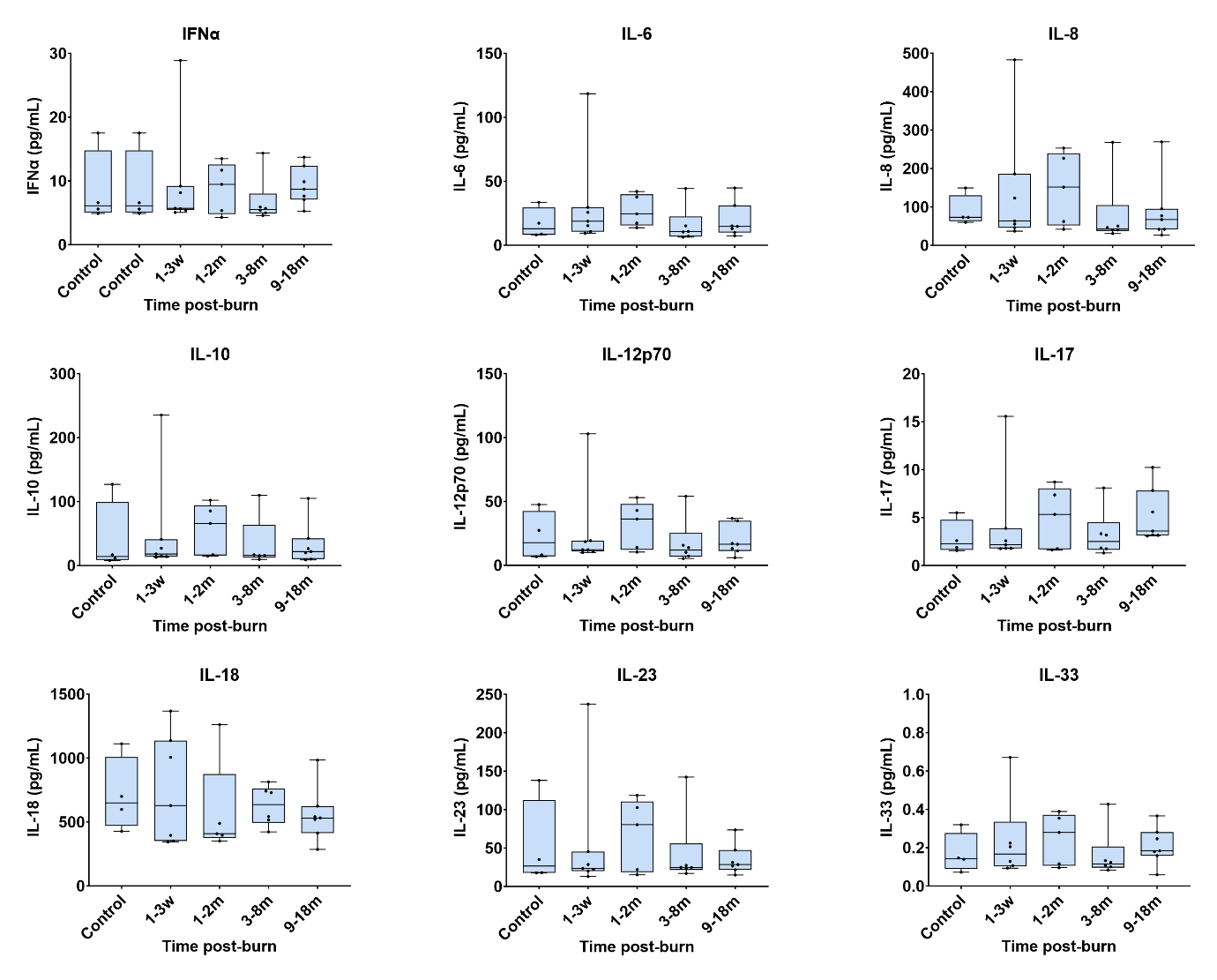


**Supplementary figure 9**. LEGENDplex Human Inflammation Panel 1 cytokine results, plasma levels of interest shown; however, they are not significant. w, week; m, month.
